# A phase I, randomized, observer-blinded, single and multiple ascending-dose study to investigate the safety, pharmacokinetics, and immunogenicity of BITS7201A, a bispecific antibody targeting IL-13 and IL-17, in healthy volunteers

**DOI:** 10.1186/s12890-018-0763-9

**Published:** 2019-01-07

**Authors:** Tracy L. Staton, Kun Peng, Ryan Owen, David F. Choy, Christopher R. Cabanski, Alice Fong, Flavia Brunstein, Kathila R. Alatsis, Hubert Chen

**Affiliations:** Genentech, Inc., 1 DNA Way, South San Francisco, CA 94080 USA

**Keywords:** IL-13, IL-17, Asthma, Anti-drug antibodies, Immunogenicity, Phase 1, Biomarker, Pharmacokinetics

## Abstract

**Background:**

Inhibition of interleukin (IL)-13, a Type 2 inflammatory mediator in asthma, improves lung function and reduces exacerbations; however, more effective therapies are needed. A subset of asthma patients also exhibits elevated IL-17, which is associated with greater disease severity, neutrophilic inflammation, and steroid resistance. BITS7201A is a novel, humanized bispecific antibody that binds and neutralizes both IL-13 and IL-17.

**Methods:**

Safety, pharmacokinetics, and immunogenicity of BITS7201A were evaluated in a phase 1 study. Part A was a single ascending-dose design with 5 cohorts: 30-, 90-, and 300-mg subcutaneous (SC), and 300- and 750-mg intravenous (IV). Part B was a multiple ascending-dose design with 3 cohorts: 150-, 300-, and 600-mg SC every 4 weeks × 3 doses. Both parts enrolled approximately 8 healthy volunteers into each cohort (6 active: 2 placebo). Part B included an additional cohort of patients with mild asthma (600-mg SC).

**Results:**

Forty-one subjects (31 active, 10 placebo) and 26 subjects (20 active, 6 placebo) were enrolled into Parts A and B, respectively. The cohort with mild asthma patients was terminated after enrollment of a single patient. No deaths, serious adverse events, or dose-limiting adverse events occurred. In Part A, 12 active (39%) and 5 placebo subjects (50%), and in Part B, 6 active (30%) and 3 placebo subjects (50%) experienced at least 1 treatment-emergent adverse event (TEAE). The most common AEs were fatigue (*n* = 3) and influenza-like illness (*n* = 2). One injection-site reaction was reported. Two subjects with elevated blood eosinophil counts at baseline had transient elevations in blood eosinophils (≥Grade 2, > 1500 cells/μL). In Parts A and B, 16 of 30 (53%) and 16 of 17 (94%) active subjects, respectively, tested positive for anti-drug antibodies (ADAs). No anaphylaxis or hypersensitivity events occurred. BITS7201A exhibited single- and multiple-dose pharmacokinetic characteristics consistent with an IgG monoclonal antibody; exposure generally increased dose-proportionally. Postdose elevations of the serum pharmacodynamic biomarkers, IL-17AA and IL-17FF, occurred, confirming target engagement.

**Conclusions:**

BITS7201A was well tolerated, but was associated with a high incidence of ADA formation.

**Trial registration:**

ClinicalTrials.gov, NCT02748642; registered April 6, 2016 (retrospectively registered).

## Background

Asthma, a chronic inflammatory disease of the airways, is a heterogeneous disease with multiple clinical and molecular phenotypes. The most commonly characterized “Type 2-high” subtype is driven by the cytokines, interleukin (IL)-4, IL-5, and IL-13. IL-4 and IL-13 promote production of immunoglobulin E (IgE), IL-5 is critical for eosinophil development and mobilization, and all three of these Type 2 cytokines are implicated in the instigation of mucus production and airway inflammation and hyperreactivity [1]. New biologics that target IL-5 signaling (anti-IL-5 [mepolizumab, reslizumab] and anti-IL-5 receptor α [benralizumab]) reduce the rate of asthma exacerbations in patients with severe eosinophilic asthma [2]. An anti-IL-13 antibody, lebrikizumab, has been shown to improve FEV_1_ (forced expiratory volume measured during the first second) and reduce IL-13-related biomarkers in clinical trials, but has not consistently shown reduction of asthma exacerbations [3, 4]. Dupilumab, a biologic that antagonizes IL-4 receptor α (IL-4Rα) signaling and thereby blocks the activity of both IL-4 and IL-13, has shown promising results, particularly in patients with elevated blood eosinophils [5, 6]. Although these treatments provide clinical benefit in subsets of patients, not all patients respond equally, suggesting that the pathways driven by these cytokines may not account for the full spectrum of disease.

IL-17, a proinflammatory cytokine expressed by cells including Type 17 T helper (Th17) cells, is also elevated in some patients with asthma and is correlated with neutrophilic inflammation, greater disease severity [7–9], and steroid resistance. Patients with both increased eosinophils and neutrophils in sputum have the lowest lung function, worse asthma control, and increased symptoms and health requirements [10]. Emerging evidence indicates that the IL-13 and IL-17 pathways are reciprocally regulated in asthma, and each contributes to the activation and recruitment of different granulocytic populations in the airway [11, 12]. Dual blockade of IL-13 and IL-17 is hypothesized to offer superior clinical benefit, particularly for patients with refractory disease. In a nonclinical, house dust mite asthma model in mice, blocking both IL-13 and IL-17 caused significant decreases in eosinophils and neutrophils in bronchoalveolar lavage fluid [11, 13].

BITS7201A is a full-length, humanized IgG4 bispecific antibody that binds and neutralizes IL-13 and IL-17 (AA, AF, and FF isoforms) in vitro and inhibits cytokine-mediated activities. In cynomolgus monkeys, BITS7201A exhibited pharmacokinetics (PK) consistent with an IgG antibody and evidence of target engagement as shown by increases in total IL-17AA (unpublished data). Although anti-drug antibodies (ADAs) were observed in the majority of animals, this was deemed to be due to the humanized form of the antibody. Nonclinical safety studies of BITS7201A supported clinical studies lasting up to 3 months.

The primary objective of this first-in-human study was to investigate the safety, PK, and immunogenicity after single and multiple doses of BITS7201A in healthy volunteers and patients with mild atopic asthma.

## Methods

### Trial design

**Fig. 1:**
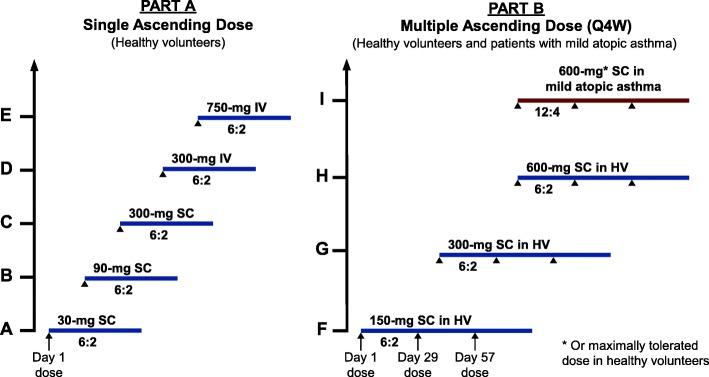
Planned dose-escalation scheme and treatment allocation (active:placebo). Because of a high proportion of ADAs in healthy volunteers, only one subject with atopic asthma was enrolled in Cohort I before study termination. HV, healthy volunteers; Q4W, every four weeks

This phase 1, randomized, observer-blinded, placebo-controlled, single and multiple ascending-dose study (ClinicalTrials.gov: NCT02748642) was conducted in two parts (Fig. 1) at a single clinical site in the United States (Knoxville, TN). Part A consisted of 5 single ascending-dose (SAD) cohorts of healthy volunteers who received the following doses of BITS7201A: (A) 30-mg subcutaneous (SC), (B) 90-mg SC, (C) 300-mg SC, (D) 300-mg IV, and (E) 750-mg IV (Fig. 1). Part B consisted of multiple ascending-dose (MAD) cohorts of healthy volunteers who each received a total of 3 doses of BITS7201A once every 4 weeks as (F) 150-mg SC, (G) 300-mg SC, or (H) 600-mg SC on Days 1, 29, and 57 (Fig. 1). The treatment allocation for these cohorts was 6 active: 2 placebo, for a planned enrollment of 40 subjects and 24 subjects in Parts A and B, respectively. Part B was to begin after safety data review of the 300-mg SC cohort in Part A. For Part B, we also planned an additional cohort of 16 patients with mild atopic asthma (Cohort I; 12 active: 4 placebo), who were to receive multiple doses of 600-mg BITS7201A SC, or the maximally tolerated dose based on the MAD, healthy volunteer cohorts.

The initial dose for BITS7201A was selected based on the no-observed-adverse-effect limit (NOAEL) of 100 mg/kg IV in a 13-week, repeat-dose cynomolgus monkey toxicity study. Safety factors were ≥ 76-fold for the proposed starting dose of 30-mg SC on the basis of body weight-based dose, human equivalent dose, maximum serum concentration (C_max_), or area under the serum concentration-time curve (AUC). The highest dose of 750-mg IV had a safety factor of at least 3-fold or greater.

### Participants

All participants were aged 18–65 years, with body mass indices (BMI) of 18–37 kg/m^2^ and weights of 50–120 kg. The study enrolled healthy volunteers as determined by medical history, 12-lead electrocardiogram (ECG), and vital signs. Patients with mild atopic asthma were required to have a diagnosis of asthma for 3 months or longer prior to screening, a history of atopy, pre-bronchodilator FEV_1_ ≥ 60% predicted at screening, and FeNO (fractional exhaled nitric oxide) ≥30 ppb at screening and randomization.

### Key exclusion criteria

Subjects were excluded if they had participated in another investigational study within 5 half-lives of the other investigational product. Because of the roles of IL-17 in the recruitment and activation of neutrophils and in immunity to extracellular pathogens such as bacteria and fungi, and because of IL-13’s role in immunity to gastrointestinal nematodes, subjects with a recent history of neutropenia or severe bacterial, fungal, or parasitic infections were excluded. Subjects with a positive tuberculin skin test or recent vaccination were also excluded. Patients with mild atopic asthma were excluded if they had symptoms consistent with poor control, active lung disease other than asthma, or occupations with potential exposure to exogenous sources of nitrous oxide and/or associated with elevated FeNO.

### Treatment assignment and blinding

The random allocation sequence was generated using SAS v.9.2 (SAS Institute, Cary, NC) by an unblinded statistician. Permuted blocks were used for each cohort. The site study team remained blinded until after database lock for final analyses, except for the independent statistician who generated the randomization list and an unblinded site pharmacist, who maintained the randomization list to ensure that eligible subjects received either BITS7201A or placebo in a 6:2 ratio for Cohorts A through H and in an approximate 12:4 ratio for Cohort I. The study pharmacist prepared and dispensed the study medication. The study subjects and site staff were blinded to treatment assignment at all times unless unblinding was required to resolve a dose-limiting adverse event (DLAE; defined below) The safety monitoring committee (SMC), composed of the medical monitor and representatives from biostatistics and drug safety, was unblinded to treatment assignments to oversee subject safety and make dose-escalation decisions.

### Study drug

The anti-IL-17 half of the antibody was derived from the parental molecule MCAF5352A (in-licensed from Novimmune SA, Geneva, Switzerland). Each half-antibody component of BITS7201A (Genentech, Inc., South San Francisco, CA) was produced in *Escherichia coli*, and the bispecific antibody was assembled from the two half-antibodies during the purification process. BITS7201A was supplied as a sterile, white, preservative-free, lyophilized powder and was reconstituted with sterile water to 150 mg/mL in 20 mM histidine hydrochloride, pH 6.0, 240 mM sucrose, 0.03% (*w*/*v*) polysorbate 20. The placebo had the same excipient composition without the active ingredient. For IV administration, study drug was diluted to 50 mg/mL in 20 mM histidine acetate, pH 5.5, 240 mM sucrose, and 0.02% polysorbate 20.

### Safety assessments and outcome measures

All subjects were confined in a clinical research unit for 24 h after each dose. In Part A, sentinel dosing was implemented for all cohorts, where the first two subjects in each cohort were randomly assigned to receive either BITS7201A or placebo, and the remaining subjects in the cohort were dosed 48 h after the last sentinel subject. All subjects were monitored for DLAEs for 14 days after dosing. Subsequent SAD cohorts would begin once all subjects in a cohort completed the safety follow-up. In Part B, Cohort F was initiated after Cohorts A–C completed Day 29, and review of ADA, PK, and safety data was complete. Subsequent MAD cohorts would be initiated after prior MAD cohorts completed safety follow-up 14 days after second dose.

Safety outcome measures included the nature, frequency, severity, and timing of treatment-emergent adverse events (TEAEs), including DLAEs, serious AEs (SAEs), and adverse events of special interest (AESIs). Changes in vital signs, physical findings, clinical laboratory, and ECG results were also monitored. AEs were coded using the Medical Dictionary for Regulatory Activities (MedDRA, version 20.0). DLAEs were defined as AEs of moderate or higher severity (≥Grade 2) that also fulfilled the following criteria: the subject had received study drug and the AE occurred within the DLAE assessment window, was clinically significant, and was related to the study drug. The following events were also considered DLAEs: allergic or hypersensitivity reactions and severe neutropenia (neutrophil count < 1000 cells/μL). AESIs were defined as: cases of potential drug-induced liver injury as defined by Hy’s Law [14]; suspected transmission of an infectious agent by the study drug; any serious hypersensitivity reaction or anaphylaxis; an injection-site reaction of Grade 2 or more; an infection of Grade 3 or more.

Immunogenicity of BITS7201A was evaluated by monitoring the incidence of ADAs and any associated clinical sequelae. In Part A, serum samples for ADA analysis were collected on Day 1 (predose), and on Days 29 (±2), 57 (±4), at any unscheduled visit, and at study completion (Day 85 ± 4) or the early termination visit. In Part B, serum samples for ADA analysis were collected on Day 1 (predose), Day 29 (±1; predose), Day 43 (±1), Day 57 (±1; predose), Day 113 (±4), at any unscheduled visit, and at study completion (Day 141 ± 4) or at the early termination visit. ADAs to BITS7201A were measured using a validated immunoassay with a relative sensitivity of 22 ng/mL and a drug tolerance of up to 100 μg/mL in the presence of 40 ng/mL of a surrogate positive control. The impact of ADA status on safety and PK was evaluated. ADA-positive subjects were defined as those who were negative for ADAs at baseline and had either treatment-induced or treatment-enhanced ADAs. Treatment-induced ADAs were defined as negative or missing baseline ADA results and at least one positive postbaseline ADA result. Treatment-enhanced ADAs were defined as positive ADA results at baseline with one or more postbaseline titer results that were at least 0.60 titer units greater than the baseline titer. Transient ADAs were defined as positive results detected at only one postbaseline sampling timepoint (excluding the last timepoint), or at 2 or more timepoints during treatment where the first and last positive samples were separated by fewer than 16 weeks. Persistent ADAs were defined as ADA-positive results detected either at the last postbaseline sampling timepoint, or at 2 or more timepoints during treatment where the first and last ADA-positive samples were separated by 16 weeks or greater.

### Pharmacokinetic outcome measures

To characterize the PK profile of BITS7201A following SC or IV dosing, serum samples were collected for Part A on Days 1, 2, 5, 8, 15, 29, 43, 57, and 85 or at early termination. For Part B, serum samples were collected on Days 1, 5, 8, 15, 29, 43, 57, 61, 71, 85, 113, and 141 or at early termination. The following PK parameters were evaluated: C_max_, clearance (CL), volume of distribution (V), AUC, half-life (t_1/2_) time to maximum serum concentration (t_max_), and bioavailability (F). Exploratory PK outcomes included assessing the relationship between drug exposure and safety and pharmacodynamic biomarkers.

### Pharmacodynamic outcome measures

Exploratory outcomes included measurement of biomarkers that could provide evidence of BITS7201A target engagement. Serum sample collection occurred in parallel with PK samples. Total serum levels of IL-17AA and IL-17FF were measured using two qualified enzyme-linked immunosorbent assays (ELISAs). The minimum quantifiable concentration (MQC) was 8 pg/mL for the IL-17AA assay and 6 pg/mL for the IL-17FF assay.

### Statistical methods

The sample size for this trial was based on the dose escalation rules and not on any statistical criteria. All statistical analyses were performed using SAS v.9.2 (SAS Institute, Cary, NC), except for PK data, which were analyzed using SAS v.9.3. Safety analyses included all subjects who received at least one dose of study drug. Safety was assessed through summaries of AEs and changes in laboratory test results, vital signs, and ECGs. All AEs were summarized by mapped term and World Health Organization severity grade.

Individual and mean serum BITS7201A concentration-versus-time data was tabulated and plotted by dose level. The pharmacokinetics of BITS7201A was summarized by estimating total exposure (AUC), C_max_, total CL (CL/F), volume of distribution (V; V/F), and t_1/2_. Bioavailability (F) was estimated using data from the 300-mg SC and 300-mg IV dose cohorts. Estimates for these parameters were tabulated and summarized (mean, standard deviation, coefficient of variation, median, range). Inter-subject variability, exposure linearity, and drug accumulation were evaluated.

For exploratory biomarker analyses, blood biomarkers were to be measured in all healthy volunteers who received at least one dose of study drug.

## Results

The study began in March 2016 and ended in June 2017, with subject enrollment termination occurring in March 2017. We planned to enroll approximately 64 healthy volunteers and 16 subjects with mild atopic asthma between the ages of 18 and 65 years, but we terminated the study after detecting ADAs in a high proportion of healthy volunteers who received BITS7201A (see Table 6). A total of 41 and 25 healthy volunteers were enrolled in Parts A and B, respectively. Only one subject with mild atopic asthma was enrolled in Cohort I prior to study termination.

In Part A (SAD), one subject in Cohort D discontinued the study due to withdrawal by subject and was replaced by an additional subject. In Part B (MAD), four subjects in the BITS7201A group discontinued the study early due to withdrawal by subject: 2 in Cohort F, 1 in Cohort G, and 1 in Cohort I. One replacement subject was enrolled in Cohort F. One subject (Cohort H) in the placebo group was lost to follow-up. Sixteen (80%) subjects in the BITS7201A group and 5 (83.3%) subjects in the placebo group completed study treatment.

The mean age of subjects in the study was 32 years (range, 18–54 years). More males (53.7%) than females were enrolled, and the majority of subjects were White (71.6%). In both Parts A and B, the demographic and baseline characteristics of the subjects were similar, with no notable differences across cohorts (Table 1). In Parts A and B, the median age of the BITS7201A group was 27 years (range, 19–54 years) and 40 years (range, 18–54 years), respectively. There was a similar proportion of males and females between treatment groups in Part A (approximately 50%), while in Part B, there were more males (55–100% in all Cohorts except Cohort H).

**Table 1:** Demographic and baseline characteristics

Part A: Single ascending-dose cohorts							
	All Placebo (*n* = 10)	All BITS7201A (*n* = 31)	Cohort A 30-mg SC (*n* = 8)	Cohort B 90-mg SC (*n* = 8)	Cohort C 300-mg SC (*n* = 8)	Cohort D 300-mg IV (*n* = 9)	Cohort E 750-mg IV (*n* = 8)
Age (years), mean (SD)	32.9 (10.75)	29.8 (9.82)	30.8 (11.41)	30.1 (10.76)	33.5 (10.64)	29.0 (10.59)	29.5 (8.40)
Sex, male, n (%),	5 (50.0)	16 (51.6)	4 (50.0)	4 (50.0)	4 (50.0)	3 (33.3)	6 (75.0)
Race, n (%)							
White	9 (90.0)	21 (67.7)	5 (62.5)	6 (75.0)	6 (75.0)	5 (55.6)	8 (100)
Black or African American	1 (10.0)	9 (29.0)	3 (37.5)	2 (25.0)	2 (25.0)	3 (33.3)	0
Multiple	0	1 (3.2)	0	0	0	1 (11.1)	0
Ethnicity, n (%)							
Hispanic or Latino	1 (10.0)	0	0	0	1 (12.5)	0	0
Height (cm), mean (SD)	172.5 (7.62)	169.7 (10.01)	170.3 (9.21)	169.8 (11.66)	169.7 (7.34)	168.2 (10.48)	174.2 (9.40)
Weight (kg), mean (SD)	77.9 (18.12)	78.8 (16.64)	80.3 (17.74)	83.3 (19.17)	75.8 (16.18)	77.2 (20.58)	76.5 (11.70)
Body mass index (kg/m^2^), mean (SD)	26.2 (5.91)	27.3 (4.98)	27.6 (5.34)	28.7 (5.25)	26.4 (5.92)	27.1 (5.65)	25.3 (4.20)
Part B: Multiple ascending-dose cohorts							
	All Placebo (*n* = 6)	All BITS7201A (*n* = 20)	Cohort F 150-mg SC (*n* = 9)	Cohort G 300-mg SC (*n* = 8)	Cohort H 600-mg SC (*n* = 8)		Cohort I^a^ 600-mg SC (*n* = 1)
Age (years), mean (SD)	32.8 (8.82)	36.1 (11.00)	33.0 (10.07)	38.1 (9.28)	35.3 (13.16)		35.0
Sex n (%)							
Male	4 (66.7)	11 (55.0)	7 (77.8)	5 (62.5)	2 (25.0)		1 (100)
Race n (%)							
White	4 (66.7)	14 (70.0)	5 (55.6)	6 (75.0)	6 (75.0)		1 (100)
Black or African American	2 (33.3)	5 (25.0)	3 (33.3)	2 (25.0)	2 (25.0)		0
Multiple	0	1 (5.0)	1 (11.1)	0	0		0
Ethnicity n (%)							
Hispanic or Latino	1 (16.7)	1 (5.0)	1 (11.1)	1 (12.5)	0		0
Height (cm), mean (SD)	169.4 (12.78)	169.8 (8.02)	169.8 (9.33)	174.1 (7.94)	164.5 (8.56)		174.0
Weight (kg), mean (SD)	84.0 (23.99)	80.8 (15.34)	75.8 (14.88)	89.6 (17.30)	76.1 (15.15)		111.0
Body mass index (kg/m^2^), mean (SD)	28.7 (4.48)	28.0 (4.90)	26.1 (3.61)	29.6 (5.24)	28.0 (4.40)		36.7

*IV* intravenous, *SC* subcutaneous

Number of subjects (n) for individual cohorts includes placebo subjects for that cohort

^a^Subject with mild atopic asthma; standard deviations (SD) were not calculated for this cohort due to *n*=1

### Safety

In Part A, 31 subjects received a single dose of BITS7201A and 10 subjects received a single dose of placebo. Twelve subjects (38.7%) in the BITS7201A group experienced 15 TEAEs compared to eight TEAEs in 5 subjects (50.0%) in the placebo group (Table 2). Treatment-related AEs were reported for 8 subjects (25.8%) in the BITS7201A group and 2 subjects (20.0%) in the placebo group. The only treatment-related AE reported more than once was fatigue. There were no serious or severe TEAEs. No TEAEs led to treatment discontinuation, and no deaths or DLAEs occurred during Part A.

**Table 2:** Part A: treatment-emergent adverse events

System Organ Class Preferred Term	All Placebo (*n* = 10)	All BITS7201A (*n* = 31)	Cohort A 30-mg SC (*n* = 6)	Cohort B 90-mg SC (*n* = 6)	Cohort C 300-mg SC (*n* = 6)	Cohort D 300-mg IV (*n* = 7)	Cohort E 750-mg IV (*n* = 6)
Total number of TEAEs	8	15	4	1	3	3	4
Number of subjects with at least 1 TEAE	5 (50.0)	12 (38.7)	2 (33.3)	1 (16.7)	3 (50.0)	3 (42.9)	3 (50.0)
General disorders and administration site conditions	1 (10.0)	5 (16.1)	1 (16.7)	0	2 (33.3)	1 (14.3)	1 (16.7)
Fatigue	0	3 (9.7)	1 (16.7)	0	1 (16.7)	1 (14.3)	0
Chills	1 (10.0)	0	0	0	0	0	0
Infusion site erythema	0	1 (3.2)	0	0	0	0	1 (16.7)
Injection site reaction	0	1 (3.2)	0	0	1 (16.7)	0	0
Infections and infestations	1 (10.0)	4 (12.9)	1 (16.7)	0	0	2 (28.6)	1 (16.7)
Chlamydial infection	0	1 (3.2)	0	0	0	1 (14.3)	0
Furuncle	0	1 (3.2)	1 (16.7)	0	0	0	0
Otitis media	0	1 (3.2)	0	0	0	0	1 (16.7)
Pharyngitis streptococcal	0	1 (3.2)	0	0	0	1 (14.3)	0
Upper respiratory tract infection	0	1 (3.2)	0	0	0	0	1 (16.7)
Viral upper respiratory tract infection	1 (10.0)	0	0	0	0	0	0
Gastrointestinal disorders	2 (20.0)	1 (3.2)	0	0	1 (16.7)	0	0
Abdominal pain upper	0	1 (3.2)	0	0	1 (16.7)	0	0
Nausea	1 (10.0)	0	0	0	0	0	0
Vomiting	1 (10.0)	0	0	0	0	0	0
Ear and labyrinth disorders	1 (10.0)	1 (3.2)	1 (16.7)	0	0	0	0
Cerumen impaction	0	1 (3.2)	1 (16.7)	0	0	0	0
Ear pain	1 (10.0)	0	0	0	0	0	0
Respiratory, thoracic and mediastinal disorders	1 (10.0)	1 (3.2)	0	0	0	0	1 (16.7)
Oropharyngeal pain	0	1 (3.2)	0	0	0	0	1 (16.7)
Rhinorrhoea	1 (10.0)	0	0	0	0	0	0
Injury, poisoning and procedural complications	0	1 (3.2)	1 (16.7)	0	0	0	0
Ligament sprain	0	1 (3.2)	1 (16.7)	0	0	0	0
Nervous system disorders	1 (10.0)	0	0	0	0	0	0
Dizziness	1 (10.0)	0	0	0	0	0	0
Psychiatric disorders	0	1 (3.2)	0	1 (16.7)	0	0	0
Libido decreased	0	1 (3.2)	0	1 (16.7)	0	0	0
Skin and subcutaneous tissue disorders	1 (10.0)	0	0	0	0	0	0
Dermatitis contact	1 (10.0)	0	0	0	0	0	0

Data are number of subjects (%). *IV* intravenous, *SC* subcutaneous, *TEAE* treatment emergent adverse event

The most common TEAEs in the BITS7201A-treated groups were general disorders and administration site conditions (5 subjects; 16.1%) and infections and infestations (4 subjects; 12.9%) (Table 2). Fatigue was reported for 3 subjects (9.7%); for 2 of these subjects, the TEAE was related to treatment. In the placebo group, gastrointestinal disorders were reported in 2 subjects (20.0%). All other TEAEs in both BITS7201A and placebo groups occurred once in other system organ classes (SOC). All TEAEs in Part A were mild and all AESIs were of Grade 1 severity and resolved.

In Part B (Cohorts F, G, and H), 3 subjects received a single dose, 2 subjects received 2 doses, and 14 subjects received 3 doses of BITS7201A. In Cohort I, one subject received 1 dose of BITS7201A. In the BITS7201A group, 8 TEAEs occurred in 6 subjects (30.0%) compared to 3 TEAEs in 3 subjects (50%) in the placebo group (Table 3). Only 1 subject (150-mg SC) in the BITS7201A group experienced a treatment-related AE. There were no serious or severe TEAEs and no TEAEs that led to treatment discontinuation. No deaths or DLAE occurred during Part B.

**Table 3:** Part B: treatment-emergent adverse events

System Organ Class Preferred Term	All Placebo (*n* = 6)	All BITS7201A (*n* = 20)	Cohort F 150-mg SC (*n* = 7)	Cohort G 300-mg SC (*n* = 6)	Cohort H 600-mg SC (*n* = 6)	Cohort I^a^ 600-mg SC (*n* = 1)
Total number of TEAEs	3	8	4	2	2	0
Number of subjects with at least 1 TEAE	3 (50.0)	6 (30.0)	3 (42.9)	1 (16.7)	2 (33.3)	0
General disorders and administration site conditions	0	4 (20.0)	2 (28.6)	0	2 (33.3)	0
Influenza like illness	0	2 (10.0)	0	0	2 (33.3)	0
Drug withdrawal syndrome	0	1 (5.0)	1 (14.3)	0	0	0
Injection site bruising	0	1 (5.0)	1 (14.3)	0	0	0
Infections and infestations	1 (16.7)	2 (10.0)	2 (28.6)	0	0	0
Otitis media	1 (16.7)	0	0	0	0	0
Strongyloidiasis	0	1 (5.0)	1 (14.3)	0	0	0
Viral upper respiratory tract infection	0	1 (5.0)	1 (14.3)	0	0	0
Nervous system disorders	2 (33.3)	1 (5.0)	0	1 (16.7)	0	0
Headache	1 (16.7)	0	0	0	0	0
Nerve compression	1 (16.7)	0	0	0	0	0
Syncope	0	1 (5.0)	0	1 (16.7)	0	0
Skin and subcutaneous tissue disorders	0	1 (5.0)	0	1 (16.7)	0	0
Erythema	0	1 (5.0)	0	1 (16.7)	0	0

Data are number of subjects (%). *SC* subcutaneous, *TEAE* treatment emergent adverse event

^a^Subjects with mild atopic asthma

The most common TEAEs in the BITS7201A group in Part B were in the SOCs of general disorders and administration site conditions in 4 subjects (20.0%) and infections and infestations in 2 subjects (10.0%; Table 3). Two subjects (10.0%) experienced TEAEs of influenza-like illness in Cohort H (600-mg BITS7201A). In the placebo group, TEAEs in the SOC of nervous system disorders were reported in 2 subjects (33.3%). All other TEAEs in the BITS7201A and placebo groups occurred once. All TEAEs were mild.

In Parts A and B, there were no clinically meaningful changes in serum chemistry, urinalysis, vital signs, or ECGs, no reports of AEs suggestive of hypersensitivity or anaphylaxis, and no AEs suggestive of immunogenicity.

Two subjects (1 subject in Cohort F [150-mg SC] and 1 subject in Cohort G [300-mg SC]) exhibited increased absolute eosinophil counts (AEC). Both subjects had elevated eosinophil counts at baseline and both were asymptomatic. A 25-year-old female in Cohort F had an AEC at screening of 200 cells/μL, 3100 cells/μL at Day 1 (before the first dose), 13,600 cells/μL at Day 57 (before the second dose) and peaked at 14200 cells/μL on Day 61. On Day 58, we found that the subject had a strongyloides infection, based on IgG positivity. We treated the subject with ivermectin, and her AEC was normal (300 cells/μL) by Day 140. The second subject, a 40-year-old male, had an increased AEC at screening of 3300 cells/μL and 2200 cells/μL on Day 1. The subject maintained a Grade 2 AEC elevation (range, 1000–3300 cells/μL) throughout the study. Due to a move, the subject withdrew consent after the first dose and terminated the study early on Day 31 with an AEC of 2400 cells/μL.

### Pharmacokinetics

**Fig. 2:**
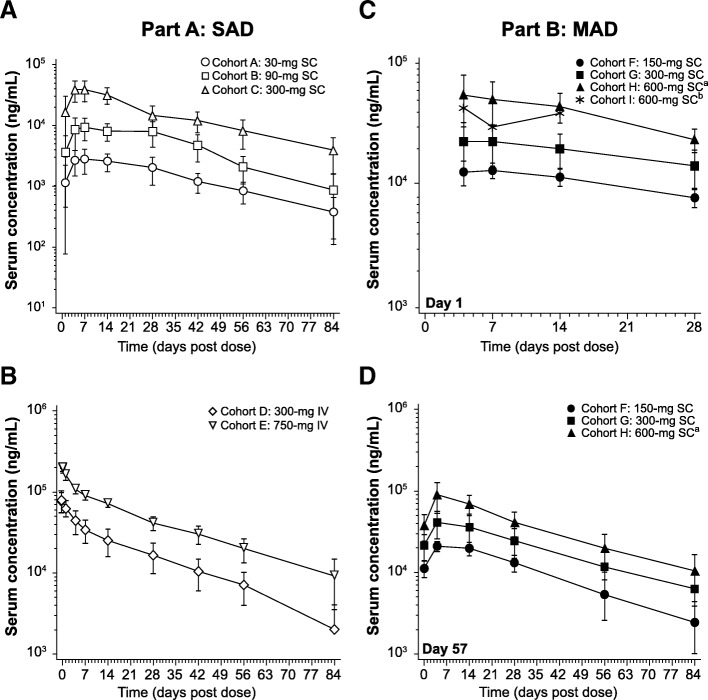
Mean (±SD) serum concentrations of BITS7201A. **a** Part A, SC cohorts. **b** Part A, IV cohorts. **c** Part B, SC cohorts after the Day 1 dose. **d** Part B, SC cohorts after the Day 57 dose. Concentrations below the limit of quantification (140 ng/mL) were set to zero. ^a^Concentration data after Study Day 15 for 2 subjects in Cohorts H were excluded due to a missed dose on Study Day 29. ^b^Cohort I: *n* = 1; data is shown only through Day 14 because only one subject was enrolled, and PK samples were not collected after Day 15

Serum concentration-time profiles for BITS7201A for both the SC cohorts and IV cohorts of Part A are shown in Fig. 2a, b, and the pharmacokinetic parameters are shown in Table 4. For SC cohorts, the median t_max_ occurred 5–7 days post dose, whereas t_max_ was achieved rapidly following the end of the IV infusion for the IV cohorts (Table 4). C_max_ and AUC_0-inf_ increased in an approximately dose-proportional manner across both SC and IV dosing cohorts in Part A. The mean terminal elimination half-life ranged from 16.4–25.5 days across all cohorts.

**Table 4:** Serum PK parameters of BITS7201A for Part A: SC and IV cohorts

Parameter	Statistics	Cohort A 30-mg SC	Cohort B 90-mg SC	Cohort C 300-mg SC	Cohort D 300-mg IV	Cohort E 750-mg IV
AUC_0-t_ (day×ng/mL)	GM mean (CV%)	110,457.2 (41.0)	370,913.6 (41.3)	1,207,764.9 (30.1)	1,176,030.6 (37.3)	3,455,348.1 (13.6)
AUC_0-∞_ (day×ng/mL)	GM mean (CV%)	141,766.6 (35.4)^a^	389,887.4 (43.5)	1,347,765.9 (32.2)	1,240,187.8 (38.6)	3,793,134.2 (18.4)
AUC% extrap. (%)	Mean (CV%)	11.09 (19.1)^a^	4.82 (66.4)	10.24 (53.6)	5.12 (69.5)	8.70 (75.1)
C_max_ (ng/mL)	GM mean (CV%)	2861.3 (37.2)	9564.4 (38.1)	39,204.9 (35.6)	77,684.8 (29.8)^b^	203,937.4 (10.3)
t_max_ (day)	Median (range)	6.973 (3.96–13.96)	6.961 (3.99–28.04)	5.495 (3.96–14.02)	0.021 (0.02–0.08)^b^	0.052 (0.02–0.08)
k_el_ (1/day)	Mean (CV%)	0.02732 (7.9)^a^	0.04404 (23.1)	0.03192 (45.4)	0.03972 (27.7)	0.03232 (37.0)
t_1/2_ (day)	Mean (CV%)	25.50 (8.1)^a^	16.42 (22.1)	24.40 (30.7)	18.60 (27.0)	23.73 (33.2)
CL/F (SC) or CL (IV) (L/day)	Mean (CV%)	0.2187 (25.5)^a^	0.2478 (39.2)	0.2452 (57.3)	0.2719 (62.2)	0.2007 (19.1)
V/F (SC) or CL (IV) (L)	Mean (CV%)	7.932 (21.0)^a^	5.663 (37.7)	7.531 (15.6)	6.956 (55.1)	6.562 (20.7)

*CV* coefficient of variation, *GM mean* geometric mean, *IV* intravenous, *SC* subcutaneous

^a^n = 5; the terminal elimination phase could not be characterized for 1 subject in Cohort A because the time spanned was less than twice the estimated t_1/2_

^b^*n* = 7; one subject discontinued early and was included in C_max_ and t_max_ calculations, but was excluded from summary statistics

*AUC*_*0-t*_ area under the concentration-time curve from time 0 to the last quantifiable concentration, *AUC*_*0-∞*_ AUC from time 0 to infinity, *AUC% extrap* AUC extrapolated to infinity as a percentage of total AUC, *CL* clearance (for IV doses), *CL/F* apparent clearance (for SC doses), *C*_*max*_ maximum serum concentration, *CV* coefficient of variation, *GM mean* geometric mean, *IV* intravenous, *k*_*el*_ elimination rate constant, *SC* subcutaneous, *t*_*max*_ time to maximum serum concentration, *t*_*1/2*_ half-life, *V* volume of distribution (for IV doses), *V/F* apparent volume of distribution (for SC doses)

Serum concentration-time profiles for BITS7201A-treated cohorts in Part B are shown in Fig. 2c and d, and the pharmacokinetic parameters after the doses administered on Days 1 and 57 are shown in Table 5. The accumulation ratio based on C_max_ ranged from 1.61 to 1.96 across cohorts, and the mean terminal elimination half-life ranged from 20.9 to 27.6 days across cohorts. PK parameters are not reported for Cohort I as only one subject was enrolled and PK samples were not collected after Day 15.

**Table 5:** Serum PK parameters of BITS7201A for Part B: SC cohorts, after dosing on Day 1 and Day 57

Parameter	Statistic	Cohort F 150-mg SC		Cohort G 300-mg SC		Cohort H 600-mg SC		Cohort I^a^ 600-mg SC
		Day 1^b^	Day 57	Day 1^b^	Day 57	Day 1^b^	Day 57^b,c^	Day 1
AUC_0-t_ (day×ng/mL)	GM Mean (CV%)	297,258.1^d^ (13.6)	835,301.5^d^ (25.8)	497,570.0^d^ (35.6)	1,602,363.7^d^ (43.1)	925,665.2 (35.6)	2,972,662.7 (31.8)	-^d^
C_max_ (ng/mL)	GM Mean (CV%)	13,551.4 (12.9)	21,512.2 (14.2)	22,502.2 (37.7)	39,102.6 (37.4)	53,814.9 (36.1)	91,308.0 (33.4)	43,100.0^e^
t_max_ (day)	Median (range)	5.481 (3.95–13.97)	3.985 (1.94–12.98)	5.929 (3.07–13.96)	3.981 (3.97–4.95)	4.053 (2.96–14.01)	4.543 (2.98–14.01)	4.949^e^
k_el_ (1/day)	Mean (CV%)		0.03240 (22.8)		0.02589 (20.5)		0.03348 (12.0)	
t_1/2_ (day)	Mean (CV%)		22.26 (21.8)		27.64 (19.4)		20.91 (12.3)	
R_ac(Cmax)_^f^	Mean (CV%)		1.607 (13.4)		1.703 (12.3)		1.956 (22.3)	

^a^Cohort *I* = 600-mg SC BITS7201A in one subject with mild atopic asthma

^b^The terminal elimination phase for the Day 1 profile for all subjects and for the Day 57 profile for one subject in Cohort H could not be characterized because the time spanned was less than twice the estimated t_1/2_

^c^Day 57 parameters for two subjects in Cohort H were excluded from the summary statistics and statistical analysis due to a missed dose on Study Day 29

^d^AUC_0-t_ for one subject each in Cohorts F, G, and I was excluded from the summary statistics due to early termination

^e^Individual value is shown

^f^R_ac(Cmax)_ = C_max_(Day 57)/C_max_(Day 1)

*AUC*_0-t_, area under the concentration-time curve from time 0 to the last quantifiable concentration, *C*_*max*_ maximum serum concentration, *CV* coefficient of variation, *GM Mean* geometric mean, *k*_*el*_ elimination rate constant, *R*_*ac(Cmax)*_ accumulation ratio based on C_max_, *SC* subcutaneous, *t*_*max*_ time to maximum serum concentration, *t*_*1/2*_ half-life

### Immunogenicity

In Part A, the baseline prevalence of ADAs was 7.7% (3 of 39 subjects). The postdose incidence of ADAs was 53.3% (16 of 30 subjects) in BITS7201A-treated subjects compared to 10% (1 of 10 subjects) in placebo-treated subjects (Table 6). ADAs were detected in Cohorts B, C, D and E, but not in Cohort A (Table 6). Out of 16 ADA-positive subjects, 1 subject developed transient, treatment-enhanced ADAs; 15 subjects developed treatment-induced ADAs, including 7 subjects with transient ADAs and 8 subjects with persistent ADAs. One of 10 subjects in the placebo group (10%) tested positive for ADAs with borderline positive signals. Six ADA-positive (37.5%) and 6 ADA-negative (42.9%) BITS7201A-treated patients experienced at least 1 TEAE, and there were no TEAEs indicating that ADAs had an effect on clinical safety. None of the TEAEs were suggestive of a hypersensitivity reaction.

**Table 6:** ADA incidence and PK parameters in ADA- and ADA+ subjects

		ADA incidence in each cohort		PK parameters			
Cohort, Dose	n	ADA-n (%)	ADA+n (%)	Elimination half-life, t_1/2_ (d), mean ± SD		Apparent clearance (L/d), mean ± SD	
Part A: SAD^a^				ADA-	ADA+	ADA-	ADA+
A, 30-mg SC	6	6 (100)	0 (0)	25.5 ± 2.1	NA	0.22 ± 0.06	NA
B, 90-mg SC	6	2 (33)	4 (67)	19.4, 21.4^c^	14.4 ± 2.3	0.24, 0.013^c^	0.28 ± 0.10
C, 300-mg SC	6	3 (50)	3 (50)	28.1 ± 5.5	20.7 ± 8.2	0.19 ± 0.03	0.30 ± 0.12
D, 300-mg IV	6	1 (17)	5 (83)	23.3^c^	18.9 ± 4.8	0.23^c^	0.29 ± 0.22
E, 750-mg IV	6	2 (33)	4 (67)	19.4, 27.4^c^	23.9 ± 9.6	0.21, 0.16^c^	0.21 ± 0.04
All BITS7201A	30	14 (47)	16 (53)				
Placebo	10	9 (90)	1 (10)				
Part B: MAD^b^							
F, 150-mg SC	5	1 (20)	4 (80)	ND	ND	ND	ND
G, 300-mg SC	6	0 (0)	6 (100)	ND	ND	ND	ND
H, 600-mg SC	6	0 (0)	6 (100)	ND	ND	ND	ND
I, 600-mg SC	n/a^d^	–	–	ND	ND	ND	ND
All BITS7201A	17	1 (5.9)	16 (94.1)				
Placebo	5	4 (80)	1 (20)				

^a^Baseline ADA prevalence (Part A, Day 1): 3 of 39 subjects (7.7%); *n* = 39 because one BITS7201A-treated subject in Cohort C did not have a Day 1 sample

^b^Baseline ADA prevalence (Part B, Day 1): 1 of 22 subjects (4.5%)

^c^For n ≤ 2, individual values are provided instead of mean ± SD

^d^Sample not available for testing

ND, not done because only 1 subject in Part B was ADA-negative

ADA-, ADA-negative; ADA+, ADA-positive

In Part B, the baseline prevalence of ADAs was 4.5% (1 of 22). The postdose incidence of ADAs was 94.1% (16 of 17) in BITS7201A-treated subjects compared to 20% (1 of 5 subjects) in placebo-treated subjects (Table 6). Of the 16 ADA-positive subjects, 15 subjects developed treatment-induced ADAs, including 4 subjects with transient ADAs and 11 subjects with persistent ADAs; 1 subject developed persistent treatment-enhanced ADAs. One of 5 (20%) placebo-treated subjects tested positive for ADAs at Day 57 only. Of 16 ADA-positive, BITS7201A-treated subjects, 5 subjects (31.3%) experienced at least 1 TEAE. The one ADA-negative, BITS7201A-treated subject and 2 ADA-negative, placebo-treated subjects experienced at least 1 TEAE. None of the TEAEs were suggestive of a hypersensitivity reaction.

Neutralizing antibodies (NAbs) were not directly measured in the study because there is no currently available assay. However, the PK profile can provide indirect evidence of the presence of NAbs. The PK assay is a target binding step-wise ELISA, and was designed to measure free BITS7201A. Presence of NAbs would likely lead to decreased concentrations of BITS7201A. We therefore examined whether the the presence of ADAs affected the PK of BITS7201A by comparing PK from ADA-positive and ADA-negative subjects. Due to a high incidence of ADAs, the sample sizes for the ADA-negative PK population were limited (Table 6). Despite this limitation, the PK from ADA-negative subjects appeared similar to the PK from the overall population for Part A (see Table 6). Additionally, a few individuals (*n* = 3) with higher ADA signals at the end of Part A had correspondingly lower serum concentrations of BITS7201A (data not shown), which possibly indicated the presence of NAbs. A similar analysis was not possible for the Part B cohorts because only a single subject was ADA-negative.

### Target engagement

**Fig. 3:**
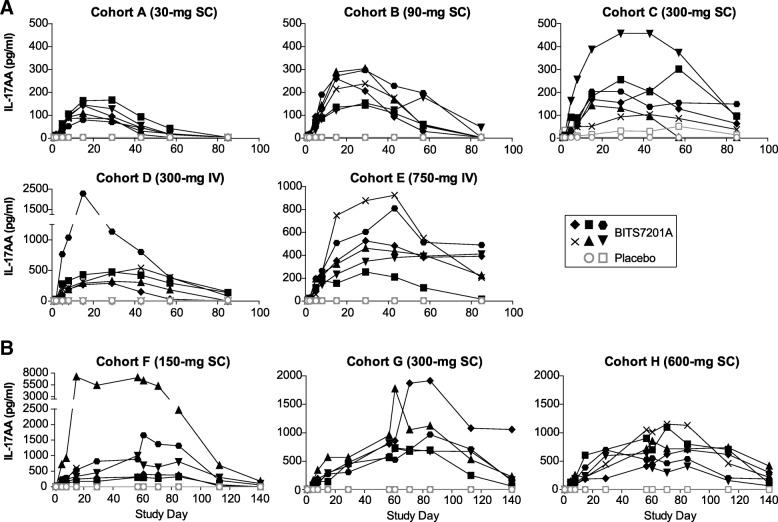
Individual subject serum levels of IL-17AA. **a** Single ascending-dose study (Cohorts A–E). **b** Multiple ascending-dose study (Cohorts F–H). BITS7201A-treated subjects, closed black symbols; placebo subjects, open gray symbols. Values that fell below the limit of detection of the assay were plotted as half of the minimum quantifiable concentration (MQC). MQC = 8 pg/mL. Some timepoints were not available for certain subjects due to subject withdrawal or loss to follow-up. IL-17AA levels were not assessed in the one patient with mild, atopic asthma in Cohort I

**Fig. 4:**
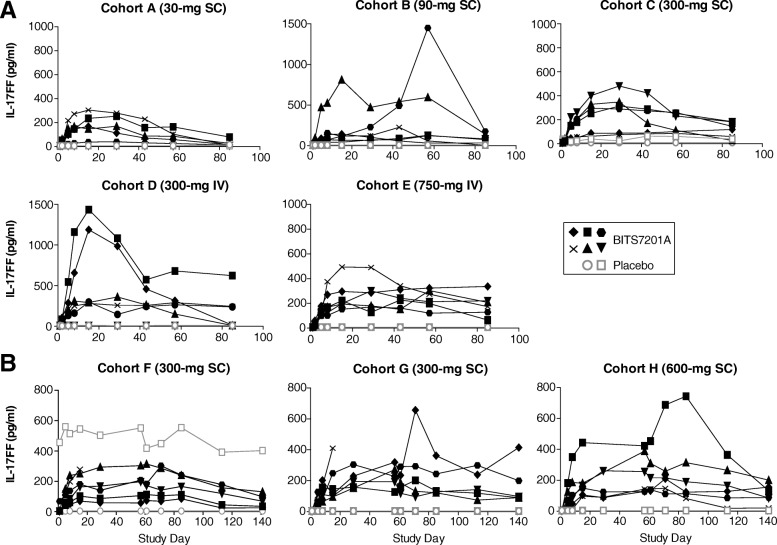
Individual subject serum levels of IL-17FF. **a** Single ascending-dose study (Cohorts A–E). **b** Multiple ascending-dose study (Cohorts F–H). BITS7201A-treated subjects, closed black symbols; placebo subjects, open gray symbols. Values that fell below the limit of detection of the assay were plotted as half of the minimum quantifiable concentration (MQC). MQC = 6 pg/mL. Some timepoints were not available for certain subjects due to subject withdrawal or loss to follow-up. One subject in Cohort F who received placebo had elevated IL-17FF at baseline and throughout the duration of the study. IL-17FF levels were not assessed in the one patient with mild, atopic asthma in Cohort I

We evaluated the pharmacodynamic effects of BITS7201A in healthy volunteers. Previous data showed that the anti-IL-13 arm in its bivalent form could sufficiently engage the target, as demonstrated by robust effects on IL-13 pathway biomarkers in asthma patients [3, 15]. To determine whether BITS7201A bound to the IL-17 isoforms, we measured serum levels of IL-17AA and IL-17FF at baseline and several timepoints post dose (Figs. 3, 4). In the majority of subjects, total serum IL-17AA and IL-17FF levels were below the assay detection limit prior to administration of BITS7201A. We found that levels of both IL-17AA and IL-17FF increased over time in the serum of subjects who received BITS7201A, reflecting antibody binding to each isoform and increasing its half-life (Figs. 3, 4). With the high affinity of BITS7201A for its target and the vast excess of BITS7201A relative to target, we assumed that virtually all IL-17AA and IL17-FF detected was bound in complex by BITS7201A and was therefore inactive. IL-17AA and IL-17FF levels did not increase in the serum of those subjects who received placebo. One subject who received placebo had elevated IL-17FF at baseline and throughout the duration of the study (Cohort F; Fig. 4). Within each cohort, the peak postdose serum IL-17AA and IL-17FF levels were variable across individuals, which may have been due to differences in predose levels of IL-17AA and IL-17FF across individuals. Although the magnitude and duration of the elevations were variable within individuals in the same cohort, in general there was a trend for increasing target engagement with increasing dose.

## Discussion

Bispecific antibodies, which can be created using different technologies, represent a relatively new class of biologic therapies. However, the safety of these molecules in humans remains relatively poorly understood compared to conventional antibody therapies. In these single and multiple ascending-dose Phase 1 studies, we investigated the safety, PK, and immunogenicity of BITS7201A, a bispecific antibody targeting IL-13 and IL-17, in healthy volunteers and a subject with mild atopic asthma. AEs were generally mild, and we observed no deaths, SAEs, or DLAEs. Although BITS7201A had satisfactory tolerability, it was associated with a high incidence of ADAs. Despite this, there were no safety events related to immunogenicity, and ADAs generally had a minimal effect on PK. PK was linear across all cohorts, consistent with a typical, stabilized IgG4 [16]. Increased serum levels of IL-17AA and IL-17FF after treatment indicated appropriate target engagement by BITS7201A.

Although prior reports have shown that immunogenicity in non-human primates is generally not predictive of human immunogenic responses [17–20], this observation was mainly based on experiences with conventional antibody therapies. We observed a high incidence of ADAs after repeated administrations of BITS7201A in cynomolgus monkeys (unpublished data), similar to humans (94% in Part B). Given the high incidence of ADAs in cynomolgus monkeys and healthy volunteers, we cannot rule out the possibility that similar events could occur in patients. In light of these clinical and preclinical data and the potential risks of administering the drug to a patient population with severe allergic asthma, we are currently conducting in vitro and in vivo studies to further characterize the root cause of the immunogenic nature of BITS7201A before considering any future clinical development.

Interpreting the clinical significance of ADAs associated with the administration of monoclonal antibodies remains a challenge. Incidences of immunogenicity vary widely, ranging from as low as < 1% to greater than 50% [21]. In general, higher incidences have been reported for older therapeutics and those utilized for the treatment of more serious conditions where the tolerance for immunogenicity may be higher. The advent of newer, humanized, and fully human monoclonal antibodies has reduced, but not eliminated, the risk of immunogenicity [22]. Among biologics approved for the treatment of asthma, the incidence of ADAs is relatively low. Antibodies to omalizumab (Xolair®) are detected in less than 1% of treated patients [23]. The incidence of ADAs in asthmatic patients treated with the anti-IL5 therapies, mepolizumab (Nucala®) and reslizumab (Cinqair®), is reported to be approximately 5% [24, 25]. Reported incidences of immunogenicity are influenced by study design, underlying disease, sampling time and frequency, sample handling, assay sensitivity and specificity, and assay susceptibility to drug interference [26], therefore making it very difficult to directly compare different therapeutics. Nonetheless, in the case of BITS7201A, after 1 to 3 doses in a healthy volunteer population, we observed an incidence of ADAs that was substantially higher than those reported for approved asthma biologics on the market.

We found two incidental cases of eosinophilia on routine laboratory testing. Both subjects had elevated absolute eosinophils at baseline prior to administration of study drug. Clinical workup of one case revealed positive serology for strongyloides (the other subject was lost to follow up). Although strongyloidiasis is uncommon in the United States, endemic foci exist in the southeastern states where the study site was located [27]. Chronic strongyloides infection can manifest itself in asymptomatic eosinophilia [27, 28]. While the observed worsening of eosinophilia in the setting of a chronic strongyloides infection could be consistent with the IL-13 blockade by BITS7201A, we cannot confirm a causal relationship, as chronic strongyloides infection itself is associated with cycles of auto-infection that can also cause asymptomatic fluctuations in eosinophil count. Notably, blocking IL-13 activity may cause increased eosinophil counts in the blood due to decreased eosinophil tracking from blood to the airways [29, 30]. In addition, rare serious adverse events associated with elevated levels of eosinophils have been described with lebrikizumab [4].

## Conclusions

In summary, our Phase 1 data demonstrate that inhibition of IL-13 and IL-17 using a bispecific antibody demonstrated acceptable safety and tolerability, but was associated with a high incidence of ADA formation. Given that only 3 monthly doses of BITS7201A were studied in healthy volunteers, we cannot exclude the potential for immunogenic consequences when it is administered as a chronic treatment in an expanded asthma patient population. Further characterization of ADAs to BITS7201A is ongoing and will contribute to our understanding of this newer class of biologic therapies.

## Abbreviations

ADA: Anti-drug antibody
AEC: Absolute eosinophil count
AESI: Adverse event of special interest
AUC: Area under the serum concentration-time curve
AUC% extrap: AUC extrapolated to infinity as a percentage of total AUC
AUC_0-∞_: AUC from time 0 to infinity
AUC_0-t_: AUC from time 0 to the last quantifiable concentration
BMI: Body mass index
CL: Clearance
CL/F: Apparent clearance
C_max_: Maximum serum concentration
DLAE: Dose-limiting adverse event
ECG: Electrocardiogram
F: Bioavailability
FeNO: Fractional exhaled nitric oxide
FEV_1_: Forced expiratory volume measured during the first second
HV: Healthy volunteer
Ig: Immunoglobulin
IL: Interleukin
IV: Intravenous
k_el_: Elimination rate constant
MAD: Multiple ascending-dose
MQC: Minimum quantifiable concentration
NOAEL: No-observed-adverse-effect limit
PK: Pharmacokinetics
ppb: Parts per billion
Q4W: Every 4 weeks
R_ac(Cmax)_: Accumulation ratio based on C_max_
SAD: Single ascending-dose
SAE: Serious adverse event
SC: Subcutaneous
SMC: Safety monitoring committee
SOC: System organ class
t_1/2_: Half-life
TEAE: Treatment-emergent adverse event
Th: T helper
t_max_: Time of maximum serum concentration
V: Volume of distribution
V/F: Apparent volume of distribution

## References

[1] Kim H, Ellis AK, Fischer D, Noseworthy M, Olivenstein R, Chapman KR, Lee J (2017). Asthma biomarkers in the age of biologics. Allergy Asthma Clin Immunol.

[2] Farne HA, Wilson A, Powell C, Bax L, Milan SJ (2017). Anti-IL5 therapies for asthma. Cochrane Database Syst Rev.

[3] Hanania NA, Noonan M, Corren J, Korenblat P, Zheng Y, Fischer SK, Cheu M, Putnam WS, Murray E, Scheerens H, Holweg CT, Maciuca R, Gray S, Doyle R, McClintock D, Olsson J, Matthews JG, Yen K (2015). Lebrikizumab in moderate-to-severe asthma: pooled data from two randomised placebo-controlled studies. Thorax.

[4] Hanania NA, Korenblat P, Chapman KR, Bateman ED, Kopecky P, Paggiaro P, Yokoyama A, Olsson J, Gray S, Holweg CT, Eisner M, Asare C, Fischer SK, Peng K, Putnam WS, Matthews JG (2016). Efficacy and safety of lebrikizumab in patients with uncontrolled asthma (LAVOLTA I and LAVOLTA II): replicate, phase 3, randomised, double-blind, placebo-controlled trials. Lancet Respir Med.

[5] Wenzel S, Castro M, Corren J, Maspero J, Wang L, Zhang B, Pirozzi G, Sutherland ER, Evans RR, Joish VN, Eckert L, Graham NM, Stahl N, Yancopoulos GD, Louis-Tisserand M, Teper A (2016). Dupilumab efficacy and safety in adults with uncontrolled persistent asthma despite use of medium-to-high-dose inhaled corticosteroids plus a long-acting β2 agonist: a randomised double-blind placebo-controlled pivotal phase 2b dose-ranging trial. Lancet.

[6] Castro M, Corren J, Pavord ID, Maspero J, Wenzel S, Rabe KF, Busse WW, Ford L, Sher L, FitzGerald JM, Katelaris C, Tohda Y, Zhang B, Staudinger H, Pirozzi G, Amin N, Ruddy M, Akinlade B, Khan A, Chao J, Martincova R, Graham NMH, Hamilton JD, Swanson BN, Stahl N, Yancopoulos GD, Teper A (2018). Dupilumab efficacy and safety in moderate-to-severe uncontrolled asthma. N Engl J Med.

[7] Lewkowich IP (2011). IL-17A in asthma − a question of severity. J Clin Cell Immunol.

[8] Chesné J, Braza F, Mahay G, Brouard S, Aronica M, Magnan A (2014). IL-17 in severe asthma. Where do we stand?. Am J Respir Crit Care Med.

[9] Lindén A, Dahlén B (2014). Interleukin-17 cytokine signalling in patients with asthma. Eur Respir J.

[10] Hastie AT, Moore WC, Meyers DA, Vestal PL, Li H, Peters SP, Bleecker ER, National Heart, Lung, and Blood Institute Severe Asthma Research Program (2010). Analyses of asthma severity phenotypes and inflammatory proteins in subjects stratified by sputum granulocytes. J Allergy Clin Immunol.

[11] Choy DF, Hart KM, Borthwick LA, Shikotra A, Nagarkar DR, Siddiqui S, Jia G, Ohri CM, Doran E, Vannella KM, Butler CA, Hargadon B, Sciurba JC, Gieseck RL, Thompson RW, White S, Abbas AR, Jackman J, Wu LC, Egen JG, Heaney LG, Ramalingam TR, Arron JR, Wynn TA, Bradding P (2015). TH2 and TH17 inflammatory pathways are reciprocally regulated in asthma. Sci Transl Med.

[12] Hart KM, Choy DF, Bradding P, Wynn TA, Arron JR (2017). Accurately measuring and modeling Th2 and Th17 endotypes in severe asthma. Ann Transl Med.

[13] Bremer M, Choy D, Jia G, Nagarkar D, Kotwal S, Lee W, Abbas A, Cai F, Arron J, Scheerens H, Staton T. Discovery of non-invasive biomarkers of IL-17 activity from experimental models of asthma. Am J Respir Crit Care Med. 2016;193:A1424. https://www.atsjournals.org/doi/pdf/10.1164/ajrccm-conference.2016.193.1_MeetingAbstracts.A1424.

[14] U.S. Food and Drug Administration. https://www.fda.gov/downloads/Guidances/UCM174090.pdf. Accessed 29 Feb 2016.

[15] Corren J, Lemanske RF, Hanania NA Korenblat PE, Parsey MV, Arron JR, Harris JM, Scheerens H, Wu LC, Su Z, Mosesova S, Eisner MD, Bohen SP, Matthews JG (2011). Lebrikizumab treatment in adults with asthma. N Engl J Med.

[16] Kamath AV (2016). Translational pharmacokinetics and pharmacodynamics of monoclonal antibodies. Drug Discov Today Technol.

[17] European Medicines Agency. Guideline on immunogenicity assessment of biotechnology-derived therapeutic Proteins 2007. Available from: http://www.ema.europa.eu/docs/en_GB/document_library/Scientific_guideline/2009/09/WC500003946.pdf. Accessed 16 Nov 2015.

[18] Ponce R, Abad L, Amaravadi L, Gelzleichter T, Gore E, Green J, Gupta S, Herzyk D, Hurst C, Ivens IA, Kawabata T, Maier C, Mounho B, Rup B, Shankar G, Smith H, Thomas P, Wierda D (2009). Immunogenicity of biologically derived therapeutics: assessment and interpretation of nonclinical safety studies. Regul Toxicol Pharmacol.

[19] van Meer PJ, Kooijman M, Brinks V, Gispen-de Wied CC, Silva-Lima B, Moors EH, Schellekens H (2013). Immunogenicity of mAbs in non-human primates during nonclinical safety assessment. MAbs.

[20] U.S. Food and Drug Administration. FDA Guidance for Industry: Immunogenicity Assessment for Therapeutic Protein Products 2014. Available from: http://www.fda.gov/downloads/drugs/guidancecomplianceregulatoryinformation/guidances/ucm338856.pdf. Accessed 16 Nov 2015.

[21] Koren E, Zuckerman LA, Mire-Sluis AR (2002). Immune responses to therapeutic proteins in humans--clinical significance, assessment and prediction. Curr Pharm Biotechnol.

[22] Harding FA, Stickler MM, Razo J, DuBridge RB (2010). The immunogenicity of humanized and fully human antibodies: residual immunogenicity resides in the CDR regions. MAbs.

[23] XOLAIR® (omalizumab) U.S. Package Insert, Genentech USA, Inc. and Novartis Pharmaceuticals Corporation (2014). Available from http://www.gene.com/download/pdf/xolair_prescribing.pdf. Accessed 16 Nov 2015.

[24] NUCALA® (mepolizumab) U.S. Package Insert, GlaxoSmithKline. 2017. Available from https://www.gsksource.com/pharma/content/dam/GlaxoSmithKline/US/en/Prescribing_Information/Nucala/pdf/NUCALA-PI-PIL.PDF. Accessed 16 Mar 2018.

[25] CINQAIR® (reslizumab) U.S. Package Insert, Teva Respiratory, LLC. 2016. Available from https://www.accessdata.fda.gov/drugsatfda_docs/label/2016/761033lbl.pdf. Accessed 16 Mar 2018.

[26] Shankar G, Arkin S, Cocea L, Devanarayan V, Kirshner S, Kromminga A, Quarmby V, Richards S, Schneider CK, Subramanyam M, Swanson S, Verthelyi D, Yim S (2014). American association of pharmaceutical scientists. assessment and reporting of the clinical immunogenicity of therapeutic proteins and peptides-harmonized terminology and tactical recommendations. AAPS J.

[27] Puthiyakunnon S, Boddu S, Li Y, Zhou X, Wang C, Li J, Chen X (2014). Strongyloidiasis--an insight into its global prevalence and management. PLoS Negl Trop Dis.

[28] Repetto SA, Durán PA, Lasala MB, González-Cappa SM (2010). High rate of strongyloidosis infection, out of endemic area, in patients with eosinophilia and without risk of exogenous reinfections. Am J Trop Med Hyg.

[29] Blanchard C, Mingler MK, McBride M, Putnam PE, Collins MH, Chang G, Stringer K, Abonia JP, Molkentin JD, Rothenberg ME. Periostin facilitates eosinophil tissue infiltration in allergic lung and esophageal responses. Mucosal Immunol 2008;1:289–96. doi: 10.1038/mi.2008.15. 10.1038/mi.2008.15 PMC2683986 19079190

[30] Johansson MW, Annis DS, Mosher DF. αMβ2 integrin-mediated adhesion and motility of IL-5−stimulated eosinophils on periostin. Am J Respir Cell Mol Biol 2013;48:503–10. doi: 10.1165/rcmb.2012-0150OC. 10.1165/rcmb.2012-0150OC PMC3653603 23306834

